# Chemokines and Cytokines as Salivary Biomarkers for the Early Diagnosis of Oral Cancer

**DOI:** 10.1155/2013/813756

**Published:** 2013-11-26

**Authors:** Gareema Prasad, Michael McCullough

**Affiliations:** Melbourne Dental School, The University of Melbourne, 720 Swanston Street, Carlton, Parkville, VIC 3010, Australia

## Abstract

Chemokines have been shown to be important in both inflammation and carcinogenesis and are able to be measured in saliva with relatively robust methods including enzyme-linked immunosorbent assays (ELISA). Thus it has been hypothesized that patients with oral cancer and oral potentially malignant lesions will have elevated levels of specific chemokines in oral fluids and that this may be used as a marker of both the early detection of malignant disease and progression to malignancy. The concept that salivary biomarkers can be easily measured and indicate disease states has profound consequences for clinical practice and may open up new strategies for the diagnosis, prognosis, and potential therapy of oral squamous cell carcinoma (OSCC). This review focuses on our understanding of cytokines and chemokines and the potential role that they may have in clinical practice.

## 1. Introduction

Oral cancer is the eleventh most prevalent cancer worldwide [1]. Oral cancers in Australia account for approximately 2-3% of all cancers and approximately 1% of all cancer deaths, with an increasing incidence over the past decades [2]. The most common oral cancer is oral squamous cell carcinoma (OSCC), which makes up 90% of all oral cancers [3], and if diagnosed early has a five-year survival rate of around 85% [4]. However, the early phase of oral cancer is often asymptomatic. Mortality for oral cancer is high because most patients seek care only when they experience late-stage symptoms (pain, persistent ulceration, unexplained bleeding, or an oral or neck mass), at which stage the disease is advanced and the survival rate decreases as low as 15–50%. Early detection of oral cancer is therefore paramount for improving survival rates and prognosis for patients with the disease.

Current diagnostic techniques focus on detection of malignant and potentially premalignant lesions in the oral cavity. Early lesions may present as unhealing lesions, mucosal colour changes, pain, tenderness or numbness, protuberances, or rough, thickened, crusted, or eroded areas [5]. Typically, premalignant and malignant lesions begin as a subtle red or white patch (erythroplakia or leukoplakia) that eventually ulcerates and progresses to an exophytic mass [6]. Regular comprehensive examinations of the oral cavity form the backbone of oral cancer screening and are especially critical in patients with identified risk habits and factors such as tobacco smoking, excessive alcohol consumption, and human papilloma virus infection [7].

The advantage of the standard visual and tactile examination is that it is simple to perform and requires no added equipment. However, subtle lesions may pass undetected, and it is difficult to make a visual distinction between benign, premalignant, and malignant lesions. Adjunctive techniques have been developed in recent years to facilitate making this distinction and enhance the effectiveness of oral examinations. Techniques such as vital staining (Toluidine Blue) and visualisation adjuncts (VELscope and ViziLite) highlight abnormal mucosa by targeting tissues undergoing rapid cell division and areas of high metabolic turnover [8]. Another adjunctive technique employs transepithelial sampling of the oral mucosa for cytologic analysis (OralCDx Brush Test system). While promising, these emergent technologies have yet to reproduce the sensitivity and specificity of examination via tissue biopsy and histopathological examination, which remains the gold standard for oral cancer diagnosis [8].

A new focus of research is the use of salivary diagnostics for early detection of OSCC, which have the advantage of being noninvasive and nontoxic. Proteins, mRNA, enzymes, and chemicals extracted from saliva have been found at sufficiently distinct levels between OSCC and control samples to be considered as potential biomarkers [9]. These biomarkers could be important indicators of physiological or pathological states and provide information for the detection of early and differential markers for disease. Salivary biomarkers offer an easy, inexpensive, safe, and noninvasive approach for disease detection [10]. They have the potential to serve as a widely available screening tool that does not rely on the localization of a lesion for diagnosis [11]. This advantage over other detection methods gives salivary biomarker screening the potential to identify patients with malignant and potentially malignant lesions.

Recent studies have assessed variation in biomarkers in patients with oral cancer. Using an array of biomarkers from oral rinses from 40 HNSCC patients and 39 controls assessed by ELISA assays, it has been shown that it is possible to distinguish HNSCC cases from controls, particularly when the patients demographics were also considered [12]. Further, extensive analyses of the plasma levels of 48 proteins (26 cytokines, 10 chemokines, and 12 growth factors) in 111 untreated OSCC patients, 112 healthy individuals, and 107 individuals with potentially malignant oral mucosal lesions showed that the levels of 12 proteins were significantly dysregulated in OSCC patients serum [13]. Furthermore, a recent extensive study had been undertaken to substantiate the development of salivary biomarkers. This study assessed a panel of putative OSCC markers in 395 subjects in 5 independent validation cohorts and found them to be independently validated, reproducible, and robust for use in a reference laboratory [14]. Thus, such studies indicate the potential of specific deregulated proteins as predictive biomarkers in oral cancer. This review will discuss the potential of cytokines and chemokines as salivary biomarkers for the early diagnosis of oral squamous cell carcinoma.

## 2. Cytokines

Cytokines are a group of small, mainly secreted proteins that affect the behaviour of cells in a diverse number of ways. The binding of cytokines to specific cell membrane cytokine receptors can induce a number of activities within the cell, such as growth, differentiation, or death [15]. Most cytokines have pleiotropic effects; however, some are generally considered as proinflammatory, such as interferon-gamma (IFN-*γ*), tumour necrosis factor-alpha (TNF-*α*), and interleukin-1beta (IL-1*β*) [16–19], whereas others are associated with anti-inflammatory effects, such as transforming growth factor-beta-1 (TGF-*β*1) [20].

Over the last 5 years, a considerable effort has been undertaken to analyse the salivary proteome. A large number of nonredundant proteins have been recognised in saliva, with one study [21] reporting over 1400 and another [22] almost 2,000, reflecting the diversity of salivary biomarker profiles that may identify and potentially aid in the management of a range of diseases [23].

Of particular interest has been the use of salivary cytokine levels as markers of both cell proliferation and oral cancer [24]. The most studied cytokines include epidermal growth factor (EGF), interleukins-6 and -8, vascular endothelial growth factor (VEGF), interleukins-4 and -10, tumour necrosis factor (TNF) and endothelin [24, 25].

Several studies have assessed interleukin-6 (IL-6), a multifunctional cytokine that participates in the inflammatory and immune responses and has been shown to promote the growth of cancer cells as well as associated with an increased rate of metastasis and an altered immune status [26–32]. Interestingly, IL-6 would appear to have different effects on different cell populations, stimulatory for some cell types while inhibitory for others [30]. IL-6 can promote tumor cell proliferation in several tumor cell lines, including human cervical carcinomas mediated cachexia [33]. In contrast, another study indicated that expression of IL-6 and its receptor can be inhibitory for cell proliferation and is correlated with good prognoses for patients with breast cancer [34]. IL-6 also has a demonstrable direct effect on cancer cells via inactivation of the p53 tumour suppressor gene as seen in human multiple myeloma cell line KAS 6/1. IL-6R overexpression was associated with larger tumors and more advanced histologic grade [35]. Irrespective of the role of IL-6, there is increasing evidence to support higher levels of IL-6 in the saliva of patients with oral cancer, as well as oral potentially malignant lesions, than in normal controls [36]. In a recent trial of 29 consecutive patients being treated for oral cancer, it was shown that patients had much higher salivary concentration of IL-6 than controls and that this concentration increased during the treatment period returning to baseline levels at discharge [36].

Other studies however have assessed a panel of pro-inflammatory cytokines as markers of malignancy [27, 37]. A recent study assessing the levels of IL-1*α*, IL-6, IL-8, VEGF-*α*, and TNF-*α* in saliva, measured using quantitative ELISA, was undertaken in a group of 18 patients with tongue SCC [38]. These salivary biomarkers were demonstrated to be increased in patients with oral cancer; significantly increased in a subgroup of patients with endophytic tongue cancer and IL-8 levels; particularly shown to correlate with poor prognosis; and intriguingly also found to be higher in control individuals who both smoked and consumed alcohol daily [38].

However, it should be noted that elevated levels of IL-6 and IL-8 have also been detected in other studies in the saliva of patients with periodontitis [39, 40]. The main limitation of this study is the relatively small sample size (*n* = 10); nevertheless, although IL-8 was found to be higher in patients with periodontitis than in healthy controls, it is detected at significantly much greater levels in patients with OSCC [41]. Yet if this is true, it should be possible to differentiate between an inflammatory process and a neoplastic process by the amount of IL-6 and IL-8. The study in 2008 by Arellano-Garcia et al. has multiple important innovative aspects, including the fact that it showed that multiplex bead based assays were as effective as ELISA assays for quantification of proteins in saliva and that IL-8 and IL-1beta were expressed at significantly higher levels in OSCC patients [41]. Although there were only 20 cancer patients, with 20 age and gender matched controls, this study nevertheless clearly indicated the potential of constituents of saliva as biomarkers for oral cancer.

A further study assessing salivary levels of TNF-*α*, IL-1*α*, IL-6, and IL-8 in a group of nine patients with OSCC with matched healthy controls [42] attempted to assess the relative influence of periodontal inflammation by using a modified gingival index to matched patients and control samples [42]. It found that IL-6 was statistically significantly higher in patients with OSCC. Interestingly though, several patients were edentulous, and thus neither they nor their matched controls would help discriminate the role of periodontal inflammation in relative salivary chemokine level, a fact compounded by the small sample size (9 patients with OSCC) [42].

Thus far then, the results of a number of studies would indicate that salivary cytokine levels are very likely to provide useful information of the presence of disease, epithelial behaviour, the local inflammatory response, and carcinogenesis. However, larger sample sized studies are required to investigate salivary cytokines and their role in the diagnosis of PML and OSCC while at the same time being able to deal with the obvious local confounding factor of inflammation and in particular periodontal disease. Further studies of the potential of a panel of salivary cytokines as a screening tool for oral cancer are apparently ongoing, the results of which are eagerly awaited as this is likely to have a profound impact on the early detection of oral cancer and thus morbidity and mortality [11]. The complexity of undertaking such a study, that would require a large number of patients who have oral cancer, patients with potentially malignant mucosal disease, and sufficient health controls as well as patients with non-neoplastic mucosal disease. This comprehensive study, thoroughly analysing the diversity of these salivary biomarkers present in health and disease, is at the same time both daunting and necessary.

## 3. Chemokines

Chemokines are a superfamily of structurally related cytokines, which share an ability to chemotactically attract their target cells along a concentration gradient [43]. It is through this ability that these molecules play an integral role in the migration of immune cells to areas of pathogen challenge. Chemokines also mediate the movement of specific cells involved in inflammatory responses that subsequently result in cellular interactions critical for mounting immune responses [43].

All chemokines are small proteins, ranging in weight from 6 to 14 KDa. There are now over 50 identified chemokines and 20 chemokine receptors [44]. Chemokines and chemokine receptors can be classified into 4 main structural families, dependent upon the position of the cysteine residues near the N-terminus. These families are the CC, CXC, C, and CX3C, with the X denoting the number of amino acids between the cysteine residues [45, 46].

Chemokines are secreted in response to signals such as proinflammatory cytokines such as interleukin (IL)-1, tumor necrosis factor (TNF), and interferon-c (IFN-c) and thus they play an important role in selectively recruiting monocytes, neutrophils, and lymphocytes [47].

Once induced, the directed migration of cells expressing the appropriate chemokine receptors occurs along a chemical ligand gradient known as the chemokine gradient. This allows cells to move toward high local concentrations of chemokines [48]. Chemokines induce chemotaxis through the activation of G-protein-coupled receptors (GPCRs), subsequently involving adhesion molecules and glycosaminoglycans (GAGs) [49]. Chemokines bind to specific cell surface transmembrane receptors coupled with heterotrimeric G proteins, whose activation leads to the activation of intracellular signaling cascades that prompt migration toward the chemokine source (chemotaxis) [50]. This interaction results in multiple signal transduction pathways being activated. One of the characteristics associated with the chemokine system is its redundancy. It has been shown that a single ligand can bind to multiple receptors and in turn a chemokine receptor may bind multiple ligands [51]. Also, many of the inflammatory chemokines have wide target cell selectivity, with some acting both on the cells of the innate and adaptive immunity [51].

The function of chemokines can be subdivided into two main families: those that are induced after inflammatory stimuli, the inflammatory chemokines, and those produced constitutively in tissues, the homing chemokines [52]. In addition to their roles in the immune system, chemokines and chemokine receptors are also involved in the pathology of a number of diseases, such as infections (e.g., HIV-1/AIDS), autoimmune disorders (e.g., psoriasis, rheumatoid arthritis, and multiple sclerosis), pulmonary diseases (asthma and chronic obstructive pulmonary disease), transplant rejection, cancer, and vascular disease [53]. Furthermore, there would appear to be significant overlap between chemokines as some of the inflammatory chemokines appear to be produced constitutively in some areas of the body [54] and some of the chemokines designated as homing chemokines can be upregulated by inflammatory stimuli [55]. For example, LARC/MIP-3*α* plays a role in both homeostatic trafficking of leukocytes, as well acting as an inflammatory chemokine during host defense [56].

Although the detection of chemokine levels by enzyme-linked immunosorbent assay (ELISA) has become a sensitive and specific method to determine the chemokine profile in patient fluids, this is not able to fully represent the actual inflammatory conditions *in vivo*. Indeed, many chemokines are posttranslationally modified by proteolytic cleavage, which can render an agonist more active or inactive or even convert the active chemokine into a receptor antagonist of the intact molecule [57].

Nevertheless, using ELISA, a recent study assessed the saliva of patients with oral cancer for the presence of both inflammatory chemokines (CXCL8, CXCL10, and CCL2), homeostatic chemokines (CXCL4, CCL14, and CCL18) [58]. Further, individuals with and without periodontitis were used as controls and it was found that H&N carcinomas give rise to a change in the chemokine composition of the oral fluid with a significant increase in CXCL8, CXCL10, and CCL14 before therapy, a finding that was not reproduced after therapy [58]. However, the levels detectable by ELISA were very low and it is likely that more refined methods could indicate not only intact chemokines, but also those modified posttranslationally [58]. These authors conclude that it can be expected that specific truncated chemokines, as well as the proteases involved in this truncation, will be linked to particular disease states. These authors postulate that proteomic analysis of biological fluids will further our understanding of the pathogenesis of specific diseases and provide solutions for new diagnostic and treatment options [58]. Since chemokines in disease can be occasionally involved in excessive recruitment of inflammatory cells, prevention of this recruitment may be an effective anti-inflammatory strategy. Furthermore, chemokine receptors are intimately involved in cellular recruitment and, along with CD4, have been shown to be an essential cofactor enabling HIV-1 viruses to infect cells [59]. Thus, in diseases that have a profound effect on the immune system, such as infection with HIV, there are various points of potential intervention that could provide anti-inflammatory and anti-HIV infectivity therapeutics, including prevention of the receptor-ligand interaction, prevention of the chemokine-glycosaminoglycan interaction, interfering with the signaling pathways that are induced upon receptor activation, and modification of receptor trafficking pathways [59].

However, these postulated potential interventions need considerable further study as the apparent redundancy in the expression of chemokines, and the overlap between homeostatic and inflammatory chemokines pathways, makes them difficult targets for diagnosis of diseases and therapeutics. In addition, it has been shown that the enhancement of the inflammatory response is aided by synergistic activity of chemokines for leukocyte migration [60]. Hence, blockage of a single chemokine may downregulate other immune responses, because of the inhibitory effect on its synergy with other chemokines [60]. Thus, there is a need to refine our ability to assess the presence of chemokines in disease states, the presence and specificity of chemokine receptors, and the specificity of functional active chemokines in specific disease states, prior to being able to define selective and specific targets for treatment.

Significant change in our understanding of the role of chemokines in OSCC has occurred in a fairly short time. A relatively early study assessed the presence of a particular chemokine (CXCL12) and its specific receptor (CXCR4), revealing that the receptor was more prevalent in oral cancers that metastasized, suggesting that this chemokine/receptor may be important in the regulation of tumour growth and organ-specific lymphatic spread [61]. A further, extensive study of 85 patients with oral SCCC utilized immunohistochemistry, RT-PCR, and western blot to assess the expression of a different chemokine, CCR7 and its ligand CCL21, in 85 patients with oral squamous cell carcinoma [10]. It was shown that CCR7 expression was positively correlated with lymph node metastasis, tumour size, and clinical stage, and these authors postulated that the interaction between this chemokine and its receptor may be significant for the induction of lymphatic spread.

The mechanism by which chemokines and chemokine receptors are involved in oral carcinogenesis has been extensively studied [19, 62–64]. CCL5 (previously known as RANTES—Regulated on Activation, Normal T cell Expressed and Secreted) has been shown to play a crucial role in migration and metastasis in human cancer cell lines and further showed that that CCL5/CCR5 axis enhanced migration of oral cancer cells, probably via MMP-9 [65]. An extensive investigation of 253 oral cancer patients, matched with 347 controls, the presence of mutations (single nucleotide polymorphisms) in the genes of specific chemokine ligands and receptors (CCL5 and CCR5) revealed an interesting dichotomy of the presence of mutations increasing risk for oral cancer while at the same time raising the potential that oral cancers with a specific chemokine profile may well have enhanced protection from metastases [66]. In a bid to rectify the dysregulated CC chemokine receptor (CCR5)/ligand, a recent study used interferon-*α*2b (IFN-*α*2b), known to upregulate CCR5 expression [67], in a small cohort of 12 oral cancer patients. These investigators showed that enhanced T-cell-mediated tumor cell killing upon IFN-*α*2b treatment and they postulate that this immunotherapy treatment may be combined with standard chemotherapy for better clinical outcome [67].

The SDF-1/CXCR4 (stromal cell derived factor 1/chemokine (C-X-C motif) receptor 4) pathway has been suggested to play a role in the metastatic dissemination of neoplasms with migration toward SDF-1 by tumor cells bearing CXCR4. Mutation in the gene of a specific chemokine receptor (CXCR4) has been noted to have an increased likelihood of more advanced oral cancer (stage III and IV by 2.66-fold) [68]. A study of 71 patients with HNSCC assessing the tissue expression levels of SDF-1 and CXCR4 found that patients with low SDF-1 had poorer metastasis-free survival (*P* = 0.026), disease-free survival (*P* = 0.006) and overall survival rates (*P* = 0.002) [69]. A recent immunohistochemical study has confirmed that this relationship showing a significant relationship between CXCL12 and CXCR4 was found both in potentially malignant lesions and oral cancer [70]. An *in vitro* experiment has recently shown that, with synthetic biology approaches, signalling selective inhibition of the CXCR4 prevented the metastatic spread of neoplastic cells [71].

Previously, investigations based on the known association between the chemokine ligand CXCL13 and prognosis of oral cancer found that the chemokine ligand/receptor axis of CXCL13/CXCR5 is not only important for cancer bone invasion and metastasis but may also be a potential therapeutic target to prevent OSCC bone invasion/osteolysis [72].

## 4. Conclusion

Current research has identified deregulated cytokines in OSCC as well as oral potentially malignant lesions, robust and reproducible methods for the assessment of these cytokines in saliva, and the possibility of a rapid salivary test as an indicator of disease and risk of malignancy. Ongoing development of such a method will have profound impact on oral cancer screening and the early diagnosis of oral cancer, potentially resulting in early treatment and a decrease in the high levels of morbidity and mortality associated with OSCC. Furthermore, there are indications that it may be possible to utilize our enhanced understanding of the chemokines associated with disease progression, metastases, and bone invasion to develop novel methods for the treatment of oral cancer.
